# Supplementary material for: Milk Quality Parameters of Raw Milk in Ecuador between 2010 and 2020: A Systematic Literature Review and Meta-Analysis
Source: Foods. 2022 Oct 25;11(21):3351. doi: 10.3390/foods11213351 (PMC9658222; doi:10.3390/foods11213351)
Supplement: Supplementary file 1 [file foods-11-03351-s001.zip › foods-1898113-supplementary.pdf]

Table S1: Information on the studies and authors used in the research

Number	Autors	Title	Year of study	Year of publication	Region	Number of samples "N"	Link
1	Albán, D; Bonifaz, N	Identificación de los puntos críticos en sistemas de producción que influyen en el conteo de células somáticas de leche cruda y en el rendimiento de queso mozzarella, Ecuador 2012	2012	2013	Inter - Andean	99	https://dspace.ups.edu.ec/handle/123456789/6048
2	Almeida, D; Bonifaz, N	Prevalencia de mastitis bovina mediante la prueba de California Mastitis Test e identificación del agente etiológico, en el centro de acopio de leche en la comunidad San Pablo Urco, Olmedo-Cayambe-Ecuador,2014	2014	2015	Inter - Andean	88	https://dspace.ups.edu.ec/handle/123456789/9834
3	Andrade, O; Ayala, L; Nieto, P; Pesántez, J; Rodas, E; Vázquez, J; Murillo, Y; Aguilar,Y; Serpa, V; Dután, J; Bustamante, J; Calle, G; Abad, V; Palacios, M	Determinación de adulterantes en leche cruda de vaca en centros de acopio, medios de transporte y ganaderías de la provincia del Cañar, Ecuador	2016	2017	Inter - Andean	475	https://publicaciones.ucuenca.edu.ec/ojs/index.php/maskana/article/view/1507/1192
4	Aroca, N; Álvarez, C	Detección cualitativa de residuos de antibióticos en leche cruda comercializada en el cantón Naranjal provincia del Guayas	2016	2016	Coast	72	http://repositorio.utmachala.edu.ec/handle/48000/7695
5	Ayala, J; Romero, H	Presencia de metales pesados (Arsénico y Mercurio) en leche de vaca al sur de Ecuador	2013	2013	Coast	20	https://revistas.ups.edu.ec/index.php/granja/article/view/17.2013.03
6	Balseca, P; Mosquera, J	Determinación cuantitativa de residuos en leche de eprinomectina usado como mosquicida en vacas lecheras	2017	2017	Inter - Andean	10	http://www.dspace.uce.edu.ec/bitstream/25000/10146/1/T-UCE-0014-003-2017.pdf

7	Buñay; N; Peralta, F; León, J	Determinación del recuento de aerobios mesófilos en leche cruda que ingresa a industrias Lacto Ochoa - Fernández Cia. Ltda	2014	2015	Inter - Andean	84	http://dspace.ucuenca.edu.ec/handle/123456789/21584
8	Cabezas, L; Cuaran, J	Influencia de las prácticas de ordeño sobre la calidad de leche de fincas ganaderas de la provincia de Pichincha	2019	2019	Inter - Andean	522	http://repositorio.utn.edu.ec/handle/123456789/9778
9	Calderón N; Morales, W	Cromatografía de AGV y células somáticas como indicadores de la calidad de leche, bajo dos sistemas de ordeño. Manabí-Ecuador	2015	2016	Coast	40	https://repositorio.uteq.edu.ec/handle/43000/2037
10	Calderón, A; Mancebo, B; Sánchez, L; Chiriboga, X; Lucero, D; Marrero, E	Niveles de ptaquilósido en muestras de leche bovina en granjas de San Miguel de Bolívar, provincia Bolívar, Ecuador	2013	2013	Inter - Andean	29	http://scielo.sld.cu/pdf/rsa/v35n2/rsa06213.pdf
11	Calderón, A; Mancebo, B; Sánchez, L; Chiriboga, X; Lucero, D; Marrero, E; Silva, J	Residualidad del ptaquilósido en la leche procedente de granjas bovinas en tres cantones de la provincia Bolívar, Ecuador	2011	2014	Inter - Andean	84	http://scielo.sld.cu/scielo.php?script=sci_arttext&pid=S0253-570X2014000100004
12	Calderón, P; Morales, W	Calidad microbiologica de la leche de bovinos de doble propósito bajo dos sistemas de ordeño en cuatro cantones de Manabí	2016	2016	Coast	40	https://repositorio.uteq.edu.ec/handle/43000/2038
13	Caracundo, E; Garnica, F	Determinación antibióticos betalactámicos y tetraciclinas en la leche cruda comercializada	2019	2019	Inter - Andean	150	https://dspace.ups.edu.ec/bitstream/123456789/17391/1/UPS-CT008305.pdf
14	Cárdenas, C; Murillo, M; Murillo, Y	Calidad Bacteriológica de la leche cruda en ganaderías de la provincia del Azuay	2018	2018	Inter - Andean	45	http://dspace.ucuenca.edu.ec/handle/123456789/31455
15	Carrasco, F	Gestión de Riesgo por presencia de residuos de antibióticos en leche cruda	2017	2017	Inter - Andean	552	http://dspace.uazuay.edu.ec/bitstream/datos/7845/1/13639.pdf
16	Carrera, M.; León, G.; Rosales, M	Mejoramiento de la calidad de la leche de pequeños productores del Cantón Biblian	2018	2018	Inter - Andean	50	http://dspace.uazuay.edu.ec/handle/datos/7817
17	Castillo ,Paola; Ortega, Renato; Vaca, Carlos	Determinación de la alteración-adulteración de leche cruda mediante análisis físico- químicos en medios de transporte legalizados, provenientes de la Parroquia Tarqui, Cantón Cuenca	2016	2016	Inter - Andean	90	http://dspace.ucuenca.edu.ec/bitstream/123456789/23505/1/Tesis%20Castillo%2c%20Ortega.pdf

18	Castro, M; Suárez, C	Determinación de la presencia de antibiótico en leche cruda de bovino comercializada directamente en la viviendas de las parroquias de Victoria del Portete y Tarqui	2017	2017	Inter - Andean	78	http://dspace.uazuay.edu.ec/bitstream/datos/6672/1/12688.pdf
19	Chacón, F.; Sagday, C.	Evaluación de los Análisis Físicos-Químicos de la Leche Bovina	2017	2017	Inter - Andean	350	https://dspace.ups.edu.ec/bitstream/123456789/13538/1/UPS-CT006912.pdf
20	Chanaluisa, J.; Quishpe, X.	Estandarización de la rutina de ordeño de bovinos en las unidades productivas del cantón Salcedo.	2018	2018	Inter - Andean	25	http://repositorio.utc.edu.ec/bitstream/27000/6065/6/PC-000523.pdf
21	Chasi, E; Bonifaz, N	Prevalencia de mastitis bovina mediante la prueba de California Mastitis Test e identificación del agente etiológico, en el centro de acopio de leche en la comunidad de Muyurco, Cayambe-Ecuador,2014	2014-2015	2015	Inter - Andean	36	https://dspace.ups.edu.ec/handle/123456789/9839
22	Chicaiza, J; Godoy, V	La calidad de la leche después del ordeño en diferentes fincas del centro sur del Cantón La Maná	2014	2015	Inter - Andean	18	https://repositorio.uteq.edu.ec/handle/43000/4439
23	Chuquimarca, A; Vayas, E	Implementación y evaluación de buenas prácticas de manufactura (BPM) y principios estándares de sanítización (SOPS) en la asociación de queseros de Guamote, para la producción de queso fresco	2009	2010	Inter - Andean	36	http://dspace.espoch.edu.ec/handle/123456789/2268
24	Chuquín, H. ; Aquino, E.; De la Cruz, E.	Diagnóstico Del Manejo De La Calidad De Leche Y Del Queso En La Provincia Del Carchi	2016	2016	Inter - Andean	399	https://revistasdigitales.upec.edu.ec/index.php/sathiri/article/view/17/34
25	Contero, R; Requelme, N; Cachipuendo, C; Acurio, D	Calidad de la leche cruda y sistema de pago de la calidad en Ecuador	2009-2018	2020	Inter - Andean, Coast and Oriental	103 204	https://lagranja.ups.edu.ec/index.php/granja/article/view/33.2021.03
26	De la Cruz, E; Simbaña, P; Bonifaz, N	Gestión de calidad de leche de pequeños y medianos ganaderos de centros de acopio y queserías artesanales, para la mejora continua. Caso de estudio: Carchi, Ecuador.	2017	2018	Inter - Andean	630	https://doi.org/10.17163/lgr.n27.2018.10

27	De la Cueva, F; Naranjo, A; Puga Torres, B; Aragón, E	Presence of heavy metals in raw bovine milk from Machachi, Ecuador	2016	2020	Inter - Andean	58	https://lagranja.ups.edu.ec/index.php/granja/article/view/33.2021.02
28	Defaz, E.; Pérez, O.; Pérez, D	Determinación De La Calidad Físico-Química Y Microbiológica De La Leche Cruda De Los Centros De Acopio De Las 10 Asociaciones Del Conlac-T	2013	2013	Inter - Andean	100	http://www.dspace.uce.edu.ec/bitstream/25000/4251/1/T-UCE-0014-60.pdf
29	Diaz, M; Fienco D	Evaluación del proceso sanitario del ordeño y control de calidad de la leche cruda procedente de los centros de acopio de las parroquias El Chaupi y El Pedregal pertenecientes al Cantón Mejía que proveen a la empresa El Ordeño.	2012	2013	Inter - Andean	60	http://www.dspace.uce.edu.ec/handle/25000/4363
30	Duy J; Garnica, F	Determinación de antibióticos betalactámicos, tetraciclinas y sulfonamidas en la leche cruda de pequeños productores	2020	2020	Inter - Andean	210	https://dspace.ups.edu.ec/bitstream/123456789/19195/1/UPS-CT008828.pdf
31	Erazo, W; Andrade, D	Estudio comparativo de la calidad de la leche cruda bovina producida en dos cantones de la provincia de Napo		2017	Oriental	68	http://dspace.udla.edu.ec/handle/33000/8164
32	Espinosa, J; Castro, B	Evaluación Mediante Citometría De Flujo De La Calidad De Leche De Los Bovinos (Bos Taurus) De Las Provincias De Pichincha Y Cotopaxi En Las Muestras Tomadas Por Pasteurizadora Quito En El Periodo Noviembre 2016- Enero 2017.	2016 - 2017	2017	Inter - Andean	1636	http://repositorio.ug.edu.ec/bitstream/redug/24825/1/T-UG-POS-DP-MBM--MBM-00048.pdf
33	Espinoza; M; Mier, J; Mosquera J	Determinación de la prevalencia de mastitis mediante la prueba california mastitis test e identificación y antibiograma del agente causal en ganaderías lecheras del cantón el Chaco, provincia del Napo	2012	2013	Oriental	174	http://www.dspace.uce.edu.ec/handle/25000/1281
34	Farinango, A; Bonifaz, N	Prevalencia de mastitis bovina mediante la prueba de California Mastitis Test e identificación del agente etiológico, en el centro de acopio de leche en la comunidad de Pulisa, Cayambe-Ecuador,2014	2014	2015	Inter - Andean	210	https://dspace.ups.edu.ec/handle/123456789/9826

35	Fonseca, L; Bonifaz, N	Prevalencia de mastitis bovina mediante la prueba de california mastitis test con identificación del agente etiológico del agente etiológico, en el centro de acopio de leche ce la comunidad el Chaupi, Cayambe – Ecuador, 2014	2014	2015	Inter - Andean	22	https://dspace.ups.edu.ec/handle/123456789/9825
36	Gonzalez, Marco; Bonifaz, Nancy	Estudio del punto crioscópico de leche cruda bovina, en dos pisos altitudinales y dos épocas del año, Ecuador 2012.	2012	2013	Inter - Andean	208	https://dspace.ups.edu.ec/bitstream/123456789/6050/1/UPS-YT00269.pdf
37	González, P; Ortín, J	Determinación de aflatoxina M1 en leche cruda de vaca en centros de acopio de pequeños productores en las cinco provincias de la sierra con mayor producción en el Ecuador	2018	2018	Inter - Andean	100	http://dspace.udla.edu.ec/handle/33000/9836
38	Guartatanga, J; Barragán, S	Diagnóstico de mastitis subclínica mediante la prueba de california mastitis test, y recuento de mesófilos (ufc) en ganaderías de la parroquia Pachicutza del cantón el Pangui	2017	2017	Oriental	68	https://dspace.unl.edu.ec/jspui/handle/123456789/18823?mode=full
39	Guevara-Freire, D; Montero-Recalde, M; Rodríguez, A; Valle, L; Avilés-Esquivel, D	Calidad de leche acopiada de pequeñas ganaderías de Cotopaxi, Ecuador	2018	2019	Inter - Andean	210	https://revistasinvestigacion.unmsm.edu.pe/index.php/veterinaria/article/view/15935
40	Heredia, D.; Simbaina, J.	Evaluation of the Quality of Bovine Milk of the Livestock Farms of Suscal, Cañar, Ecuador	2019	2019	Inter - Andean	60	https://pdfs.semanticscholar.org/c94b/bf8662571a1161534a39c08dc686494eb37b.pdf?_ga=2.216568670.917894491.1617161864-1895406698.1617161864
41	Jiménez, Diana; Bahamonte, Raúl	Estudio de la adulteración de leche cruda con suero de quesería, mediante cromatografía líquida de ultra eficiencia (uplc).	2015	2015	Inter - Andean	8	http://www.dspace.uce.edu.ec/bitstream/25000/6418/1/T-UCE-0008-089.pdf
42	Lagla, M; Albuja, A	Evaluación Higiénico-Sanitaria De La Quesera Artesanal Cod.Q 7 Ubicada En El Cantón Mocha, Provincia Tungurahua	2018	2018	Inter - Andean	90	http://dspace.espoch.edu.ec/bitstream/123456789/9022/1/56T00809.pdf

43	Malla, A; Saula, S; Uguña, M	Determinación del metabolito tóxico Aflatoxina M1 en leches cruda, pasteurizada y ultrapasteurizada consumidas en la ciudad de Cuenca mediante la técnica de Cromatografía Líquida de Alta Resolución (HPLC)	2015	2015	Inter - Andean	4	https://dspace.ucuenca.edu.ec/bitstream/123456789/23399/1/Tesis.pdf
44	Marañón, J.; Reina, J	PARÁMETROS DE CALIDAD EN LECHE CRUDA SEGÚN LA NORMA NTE INEN 0009:2012 EN CENTROS DE ACOPIO DE LA PROVINCIA DE SANTO DOMINGO DE LOS TSÁCHILAS	2017	2017	Coast	24	https://repositorio.espe.edu.ec/bitstream/21000/12959/1/T-ESPE-002799.pdf
45	Martínez-Villarreal, D; Morales, S; Núñez, L; Santander, S; De la Cueva, F; Puga-Torres, B	Determination of the hygienic and physico-chemical quality of raw milk produced by small and medium producers of the North-East region of Carchi-Ecuador	2017	2017	Inter - Andean	694	http://www.dspace.uce.edu.ec/bitstream/25000/14985/1/Determination%20of%20the%20hygienic%20and%20physico-chemical%20quality%20of%20raw%20milk%20produced%20by%20small%20and%20medium%20producers%20of%20the%20north-east%20region%20of%20Carchi%20-%20Ecuador.pdf
46	Mera, P	Evaluación De La Calidad De La Leche Mediante Citometría De Flujo, Proveniente De Bovinos De La Parroquia Machachi, Provincia De Pichincha.	2013	2013	Inter - Andean	421	http://repositorio.espe.edu.ec/handle/21000/7465
47	Monge, R; Cuarán, M	Diseño de un centro de acopio modelo para leche cruda	2017	2017	Inter - Andean	9	http://repositorio.utn.edu.ec/bitstream/123456789/7773/1/03%20EIA%20447%20TRABAJO%20DE%20GRADO.pdf
48	Moreira, E.; García, R.; Montesdeoca R.; Buste, M.; López, G.	Diagnóstico de la calidad higiénico sanitaria de la leche de los sistemas bovinos del Cantón El Carmen	2019	2020	Coast	5	http://www.revistaecuatorianadecienciaanimal.com/index.php/RECA/article/view/197/160
49	Mosquera, J; León, P	Diseño de un sistema de buenas prácticas de ordeño basado en la resolución MAGAP-Agrocalidad N° 0217 para la hacienda San José del Belén en el sector de Tambillo	2019	2019	Inter - Andean	10	http://repositorio.puce.edu.ec/bitstream/handle/22000/17301/proyecto%20Xavier%20Mosquer

							a%20f.pdf?sequence=1&isAllowed=y
50	Neppas, E.; Requelme, N	Sistematización y análisis del proceso de gestión de la calidad de la leche del centro de acopio "El Progreso" de Cariacu, cantón Cayambe	2010-2011	2014	Inter - Andean	149	https://dspace.ups.edu.ec/handle/123456789/7514
51	Ortíz, Maira; Cazar M	Determinación de la presencia de Aflatoxina M1 y Antibióticos en leche cruda de las fincas de mayor producción del cantón Biblán	2014	2014	Inter - Andean	88	http://dspace.uazuay.edu.ec/bitstream/datos/3341/1/10109.pdf
52	Ortíz, Maira; Rosales, Cornelio; Aguilar, Yolanda; Murillo, Yuri; Serpa, Guillermo; Paguay, Tatiana; Coronel, Ángel	Estudio exploratorio sobre la presencia de contaminantes en leche cruda proveniente de la cuenca lechera del Tarqui de la Sierra Sur Ecuatoriana	2016	2017	Inter - Andean	90	http://dspace.ucuenca.edu.ec/bitstream/123456789/27692/1/MASKANA%208111.pdf
53	Paguay, T; Coronel, A; Ortíz, M	Determinación de la incidencia de adulterantes e inhibidores de leche cruda almacenada en diez centros de acopio de la Provincia del Azuay	2015	2015	Inter - Andean	90	http://dspace.ucuenca.edu.ec/handle/123456789/23504
54	Peña, F; Cuarán, M	Diseño de un manual de buenas prácticas de manufactura para centros de acopio de leche cruda	2019	2019	Inter - Andean	57	http://repositorio.utn.edu.ec/handle/123456789/9775
55	Pérez, M; País, J	Evaluación de la calidad higiénica sanitaria de leche cruda mediante la prueba de lactofermentación a nivel de centros de acopio en la provincia del Carchi	2016-2017	2019	Inter - Andean	616	http://repositorio.utn.edu.ec/handle/123456789/8828
56	Pilamunga, C; Albuja, A	Evaluación Higiénico – Sanitaria De La Quesera Artesanal Cod.Q 1 Ubicada En La Parroquia Químiag Del Cantón Riobamba, Provincia De Chimborazo	2017	2017	Inter - Andean	3	http://dspace.espoch.edu.ec/handle/123456789/6937
57	Puga, B; Cáceres, M; Alarcón, D; Gómez, C	Determination of Zearalenone in raw milk from different provinces of Ecuador	2019	2021	Inter - Andean and Coast	209	www.doi.org/10.14202/vetworld.2021.2048-2054
58	Puga, B; Salazar, D; Cachiguango, M; Cisneros, G; Gómez, C	Determination of Aflatoxin M1 in Raw Milk from different provinces of Ecuador	2019	2020	Inter - Andean and Coast	209	https://www.mdpi.com/2072-6651/12/8/498

59	Rivera, N; Rodríguez, S; Pérez, C	Diagnóstico de situación del proceso productivo y evaluación de la calidad de la leche en la Asociación Agropecuaria "El Trébol"	2018	2019	Inter - Andean	59	http://www.dspace.uce.edu.ec/bitstream/25000/18314/1/T-UCE-0014-MVE-044.pdf
60	Rodríguez, A; Montero, M	Determinación De La Inocuidad Y Calidad Fisicoquímica De Leche Cruda En Plantas Procesadoras Del Cantón Salcedo	2016	2016	Inter - Andean	30	https://repositorio.uta.edu.ec/jspui/handle/123456789/24354
61	Sánchez, C; Rojas, 2013	Estudio preliminar de Aerobios Mesófilos en la leche cruda que se expende en carros repartidores en la ciudad de Cuenca	2013	2013	Inter - Andean	82	http://dspace.uazuay.edu.ec/handle/datos/3206
62	Romero, J; Pincay, P	Evaluación de la calidad fisicoquímica y microbiológica de la leche cruda obtenida de dos haciendas ubicadas en el cantón Bucay provincia del Guayas	2016	2016	Coast	96	http://repositorio.ucsg.edu.ec/handle/3317/6933
63	Rosero, D; Chiriboga, X	Extracción, identificación, cuantificación de Ptaquilósido en leche de ganado vacuno que pastorea en zonas donde crece Pteridium arachnoideum	2013	2013	Inter - Andean	15	http://www.dspace.uce.edu.ec/bitstream/25000/1903/1/T-UCE-0008-19.pdf
64	Salguero, A; Puga, B	Calidad de leche cruda de pequeños productores del cantón Cayambe y Pedro Moncayo, por análisis físico químicos y ensayos cualitativos	2021	2019	Inter - Andean	132	http://www.dspace.uce.edu.ec/handle/25000/20256
65	Salvador, J; Peñafiel, J	Determinaciónde la incidencia de mastitis subclínica mediante los métodos California Mastitis Test (CMT) y Somaticell en cinco ganaderías del cantón Vinces provincia de Los Ríos	2010-2011	2011	Coast	1176	http://repositorio.ucsg.edu.ec/handle/3317/993
66	Torres, M; Díaz, M	Determinación de niveles de tetraciclina y oxitetraciclina en leche cruda en la asociación copla (corporación productora de leche de Alóag) de la parroquia Alóag del cantón Mejía	2015	2015	Inter - Andean	8	http://www.dspace.uce.edu.ec/bitstream/25000/6427/1/T-UCE-0008-106.pdf
67	Valle, T.; Díaz, B.	Evaluación De La Calidad De La Leche Cruda E Implementación De Un Manual De Calidad En El Centro De Acopio: Asociación El Panecillo, Tungurahua	2015	2015	Inter - Andean	24	http://dspace.espoch.edu.ec/bitstream/123456789/4621/1/56T00600%20UDCTFC.pdf

68	Vallejo, C; Díaz, R; Morales, W; Godoy, V; Calderón, N; Cegido, J	Calidad físico-química e higiénico sanitaria de la leche en sistemas de producción doble propósito, Manabí-Ecuador	2017	2018	Coast	40	https://talentos.ueb.edu.ec/index.php/talentos/article/view/28/35
69	Vallejo, J; Ramón, E	Influencia del ordeño en el recuento de células somáticas sobre la calidad del queso andino en la organización inti Churi"	2021	2020	Inter - Andean	28	http://dspace.ueb.edu.ec/handle/123456789/3629
70	Velastegui, C; Proaño, D	Evaluación del sistema de gestión de calidad de la leche en unidades productivas y centros de acopio del cantón Quito	2017	2018	Inter - Andean	86	http://dspace.udla.edu.ec/handle/33000/9206
71	Verdesoto, V; Carrasco, W	El ordeño manual en bovinos de leche y su incidencia en la contaminación microbiana en la parroquia Quinchicoto, Cantón Tisaleo - Tungurahua	2015	2015	Inter - Andean	60	http://dspace.ueb.edu.ec/handle/123456789/1283
72	Vilegas, N. ; Díaz, J. ; Hernández, A.	Evaluación de la eficiencia tecnológica en la elaboración artesanal de queso fresco de coagulación enzimática	2016	2017	Inter - Andean	28	http://scielo.sld.cu/scielo.php?script=sci_abstract&pid=S2224-61852017000300002&lng=es&nrm=iso
73	Villegas, Z. ; Freire, J.l Yépez, L	Evaluación de la calidad físico química y microbiológica de la leche cruda que se expende en el Cantón Bolívar Provincia del Carchi	2010	2011	Inter - Andean	36	http://repositorio.utn.edu.ec/handle/123456789/386
